# Prospective study of 158 adult scoliosis treated by a bivalve polyethylene overlapping brace and reviewed at least 5 years after brace fitting

**DOI:** 10.1186/s13013-016-0091-x

**Published:** 2016-10-14

**Authors:** Jean Claude de Mauroy, Cyril Lecante, Frédéric Barral, Sophie Pourret

**Affiliations:** 1 Department of Orthopaedic Medicine, Clinique du Parc, 155 Boulevard Stalingrad, 69006 Lyon, France; 2 Orten, 125 Rue Bataille, 69008 Lyon, France

## Abstract

**Background:**

The conservative orthopaedic treatment of adult scoliosis is very disappointing. In a series of 144 patients; only 25 % (33 cases) were monitored at 2 years of treatment. (Papadopoulos 2013). Thereby the literature typically focuses on a small number of patients, which limits the usefulness and relevance of its results. The brace effect on pain has been systematically described, but there is no publication on the effect of treatment on the Cobb angle and main clinical parameters.

**Methods:**

From a prospective database started in 1998, we selected all 158 consecutive patients effectively treated conservatively with the Lyon management treatment and controlled five years after brace fitting. Lyon management includes a lordosing bivalve polyethylene overlapping brace in association with specific physiotherapy. The brace can either be short with anterior support under the chest or long with sterno-clavicular support when there is a high thoracic kyphosis.

**Results:**

1. For the rate of scoliosis controlled after 5 years, the follow-up was 24 % of the 661 patients accepting the treatment. Pain is almost the main reason for the medical consultation, generally correlating with an increase of the scoliotic angulation.

2. The descriptive data can be superimposed on general group with age (*m*=56 years, SD=13) but initial Cobb angulation is significantly higher (*m*=40°, SD=17). Ratio Female/Male=0.91.

Generally, the scoliosis is stabilized at (*m*=39.74 °, SD=19.40), 8 years after the beginning of the treatment.

38 improvements of more than 5°= 24 %; 88 stable = 56 %; 32 worsening of more than 5° = 20 %

The rib hump is improved of by 3 mm, (modelling effect of the brace).

The occipital axis is improved by more than 6 mm.

But the T1 plumb line distance is worsening by 7 mm (most braces are short without sterno-clavicular support).

**Conclusions:**

For the first time, the number of records and follow up after 8 years allows to study the radiological progression of adult scoliosis rigid bracing. Stability or improvement of more than 5° in 80 % of cases justify rigid bracing in adults. The accentuation of the thoracic kyphosis is the only negative element and a modified ARTbrace will soon be used.

**Electronic supplementary material:**

The online version of this article (doi:10.1186/s13013-016-0091-x) contains supplementary material, which is available to authorized users.

## Background

Scoliosis is a major demographic health issue in the adult population with pain, imbalance and curve angular progression. The natural evolution of late onset scoliosis has been well described with annual progression of 1° for curves of more than 50° [1, 2]. Surgeons are often very conservative in the treatment of adult scoliosis because of the complication rates higher than for adolescent and the marginal bone quality endemic to this population. There is currently a lack of literature for adult scoliosis rigid bracing. Rare publications present the short term results on pain and some examples of rigid bracing [3–5].

A 5-year minimum follow up can help to quantify the effect of the brace on the angular progression and other clinical parameters of adult scoliosis like bib hump, frontal and sagittal balance.

## Methods

The Lyon Conservative treatment requires:

1. A plaster cast made in a specific standing frame for 3 weeks.
2. A rigid polyethylene bivalve overlapped brace worn for at least 4 h per day.
3. A specific physiotherapy to prevent muscle atrophy.

The plaster cast or full time bracing is an indispensable prerequisite for this treatment. Besides the therapeutic role of muscular-ligamentous adjustment of paravertebral tension (creep), it can also be used as a test. The patient must be pain-free while pursuing normal activities [6].

The principle of bracing is completely different from that of adolescent scoliosis. Indeed, the aim is to:

1. Decompress the discs with the “hourglass effect” lifting the trunk under the fluctuating ribs and transfer the pressure on the iliac crest.
2. Rebalance the spine in both frontal plane and sagittal plane, mostly by recreating lumbar lordosis.
3. Relieve pain by the analgesic effect of rigid low back brace.

**Fig. 1:**
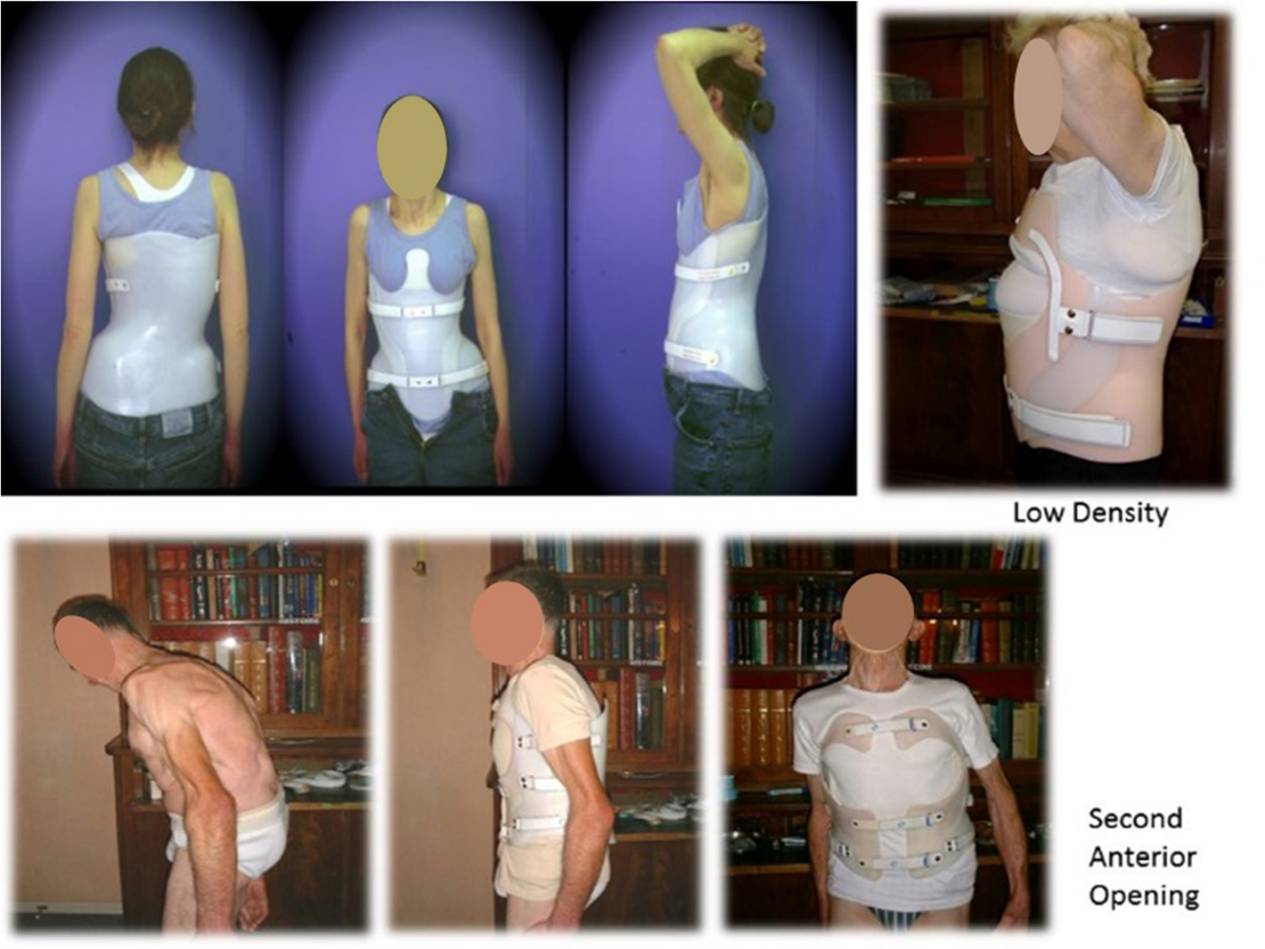
Some samples of polyethylene bivalve overlapping braces. The anterior thrust can be xyphoidal under the breast or sternoclavicular. It is performed after correcting plaster cast

The protocol is full time 24 h a day during 3 weeks, and at least four hours per day for a minimum of 6 months (Fig. 1).

## Results

### Group definition

From 1998 to 2013, rigid bracing with plaster cast for adult scoliosis was proposed in 739 cases (group A). 661/739 = 83 % patients: have accepted the treatment. 158/739 = 21 % were reviewed at least 5 years after the start of treatment (group B) (Additional file 1 – Excel spreadsheet with results of 158 patients).

SPSS 20 pack with a Confidence interval of 95 % is used (Table 1).

**Table 1:** Comparison of 8-year follow-up group with all patients

Group A	Age = 56.94±15.82	Cobb = 35.58±16.76	739
Group B (8 years follow-up)	Age = 56.08±17.35 ns(*p*=0.525)	Cobb = 39.67±17.35 (*p*=0.007)*	158

The same age at the beginning of treatment, but Cobb angle significantly higher (4 °/35 °)(* = significant P value)

There is no statistical difference in age between the two groups, but the initial angulation is significantly 4 ° higher.

Female/Male ratio is 91 %. The average follow-up is 8.41 years ± 3.26 (from 5 to 17 years).

### Descriptive statistics

Four parameters are studied: 1. Cobb angulation, 2. frontal balance with C7 plumb line, 3. sagittal balance with C7 plumb line, 4. Rib hump measured in mm (Table 2).

**Table 2:** Comparing means at the beginning and at the last control with *T*-Test

	Initial	Final follow-up	*T* test
Cobb angle	*m* = 39.64 ± 16.76	*m* = 39.74 ± 19.46	*p* = 0.973 (ns)
Frontal Balance	*m* = 16.79 ± 20.34	*m* = 10.44 ± 21.54	*p* = 0.008*
Sagittal balance	*m* = 58.04 ± 45.52	*m* = 64.72 ± 52.62	*p* = 0.231 (ns)
Rib hump (mm)	*m* = 22.88 ± 13.65	*m* = 20.06 ± 14.13	*p* = 0.073 (ns)

No significant difference for Cobb angle, sagittal balance and rib hump, while one could expect a worsening, only the frontal balance is significantly improved(* = significant P value)

The Cobb angle has been stabilized by bracing. The average is the same when it should be greater by 4° (0.5 ° × 8 years) if we take into account the spontaneous natural evolution of scoliosis in adulthood.

**Fig. 2:**
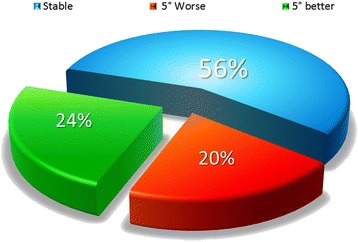
Cobb angle results with SRS criteria. 80 % of scoliosis are stable or improved more than 5°

Taking into account the SRS criteria to express the results, we get the following results:

1. Stable: 88/158 = 56 %
2. Improvement of more than 5 °: 38/158 = 24 %
3. Worsening of more than 5 °: 32/158 = 20 % (Fig. 2)

Clinical frontal balance is significantly improved (6mm).

Although non statistically significant, clinical sagittal balance is worsened 6mm.

**Fig. 3:**
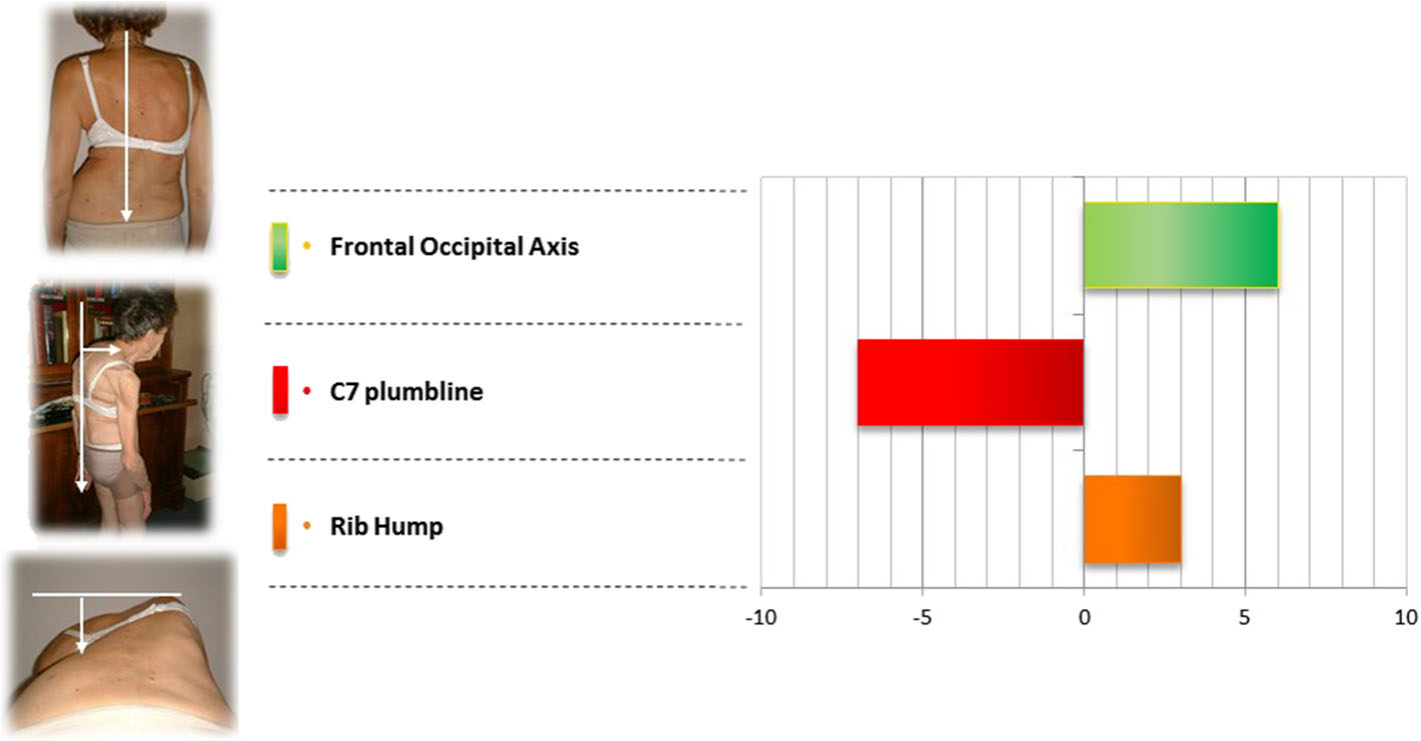
Clinical parameters results. Significant improvement of the frontal occipital axis, worsening of sagittal imbalance and stability of the rib hump

There is no significant difference at the clinical rib hump, which is favourable because the spontaneous evolution would be in the direction of worsening (Fig. 3).

The analysis shows that 18 scoliosis have increased by more than 10°, in 8 cases, the brace was no longer worn, but in 10 cases, it was worn at least 4 h a day. It is, therefore, true that there are failures. It’s not only a compliance problem as in 28 cases, the brace is not worn and the scoliosis remained stable.

There was also 1 suicide, but as a result of breast cancer.

## Discussion

**Fig. 4:**
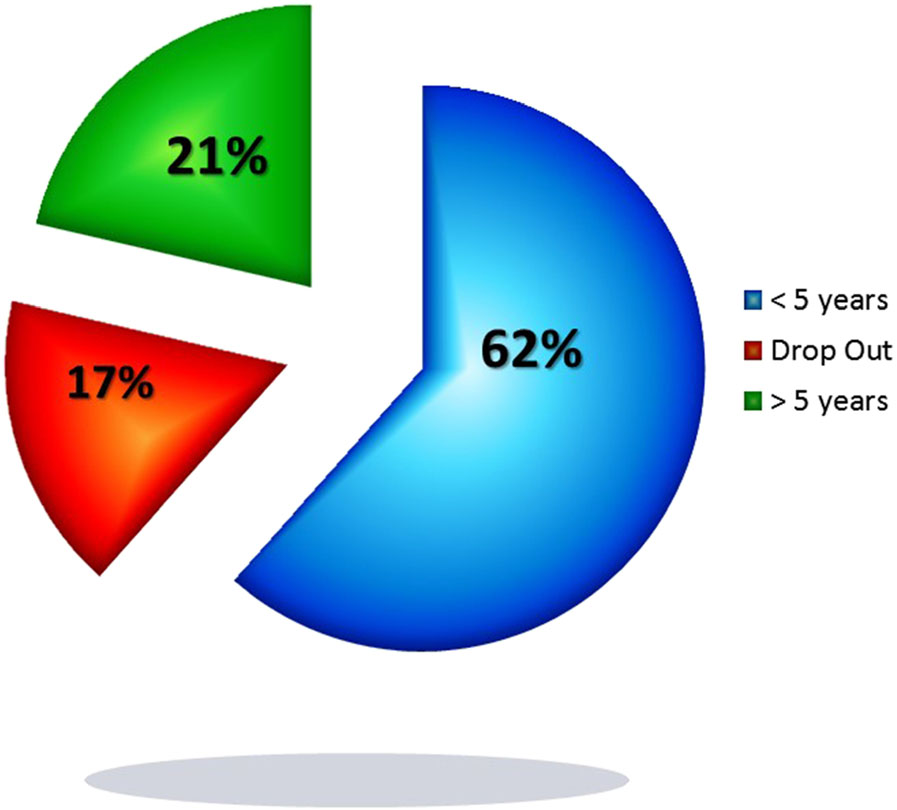
Distribution of 739 adult scoliosis with rigid bracing indication. Non-adherent patient’s rate is 17 %. 21 % of patients were monitored 5 years after the start of treatment

Bracing adult lumbar and thoracolumbar painful and instable scoliosis with polyethylene bivalve overlapping brace is effective. The aim of the treatment is a disk protection and a three-dimensional re-equilibration of the spine. We have shown in preliminary work that this treatment despite the plaster cast is well accepted with only 17 % of drop out (Fig. 4).

As in all publications, pain is improved by wearing the brace, but bracing is not only palliative, it’s a real treatment of lumbar instability mainly by discharging the pressure in the disc and stabilizing the lumbar area in lordosis to restore the tensegrity of the spine.

The results show that 8.5 years after the beginning of treatment, the natural angular evolution of scoliosis is halted in 80 % of cases.

However, it appears that current braces fail to stop the kyphotic evolution of adult scoliosis and justifies the improvement of existing braces.

Despite the undeniable technical progress, all adult scoliosis do not have an indication for surgery and adult rigid bracing appears to be a reliable alternative.

## Conclusions

Adult rigid bracing is not only a pain killer. When treatment is carried out rigorously, it can stabilize the evolution of scoliosis in 80 % of cases during 8 years. Frontal balance is significantly improved. Although not significant, sagittal balance worsened and justifies the use of new braces.

## Additional file

Additional file 1: Excel spreadsheet with results of 158 patients. Data from the prospective database (age, Cobb, balance, follow up at the Beginning and last follow-up). (XLSX 24 kb)
